# DRUG DEVELOPMENT AND EVALUATION: AGE, SEX, AND FRAILTY CONSIDERATIONS TO FACILITATE THE FORTITUDE FACTOR

**DOI:** 10.1093/geroni/igae098.1644

**Published:** 2024-12-31

**Authors:** Sarah Hilmer

**Affiliations:** Royal North Shore Hospital, Sydney, Australia, Sydney, New South Wales, Australia

## Abstract

Development and evaluation of drugs used by older adults must consider the effects of ageing, of lifelong factors including sex, and of other characteristics common in geriatric patients that affect pharmacokinetics and pharmacodynamics and outcomes, such as frailty. These factors interact within older people, resulting in major inter-individual variability. In some cases, the intersection of age, sex, frailty and other factors act in the same direction, e.g, age, female sex and frailty may all reduce renal drug clearance. In others they may act in opposite directions, e.g. age may decrease pharmacodynamic sensitivity to a drug, male sex may increase it, and frailty may reduce reserve to tolerate drug effects. Interactions with the older person’s other drugs in the presence of polypharmacy, their other diseases with multimorbidity, their genetics and environmental exposures further complicate prediction of drug effects in older adults. The International Union of Basic and Clinical Pharmacology (IUPHAR) Geriatric Committee’s position statement on ‘Measurement of Frailty in Drug Development and Evaluation’ outlines key principles relevant to all phases of translational research. These include measurement of frailty, testing drug exposures and outcomes that are clinically relevant for older adults, use of physiologically based pharmacokinetic-pharmacodynamic modelling and artificial intelligence. In all phases of drug development and evaluation, the pre-clinical models used and clinical trial populations studied should be representative of the people who will use the drugs in age, sex, frailty, and other determinants of drug response. This will inform drug use that facilitates the fortitude factor in older adults.

